# Eating on camera: a creator-centric case study of mukbang engagement in India

**DOI:** 10.3389/fpsyg.2026.1806358

**Published:** 2026-07-08

**Authors:** Gayatri Batra, Paweł A. Atroszko, Gayatri Kotbagi

**Affiliations:** 1 Department of Psychological Sciences, FLAME University, Pune, India; 2 Institute of Psychology, Faculty of Social Sciences, University of Gdańsk, Gdańsk, Poland

**Keywords:** algorithmic culture, autonomous sensory meridian response (ASMR), creator centric, digital commensality, eating behaviors, mukbang, vicarious eating

## Abstract

Mukbang, a global digital phenomenon in which individuals consume large quantities of food on camera, has gained increasing popularity across cultural contexts. Despite its growth in India, limited research has examined mukbang through the perspective of content creators. The present study addresses this gap through an in-depth qualitative case study of an Indian mukbanger and male fitness content creator from Uttarakhand (“A”). A 75-min semi-structured interview was conducted via Zoom with an Indian mukbanger. The analysis generated three interconnected themes reflecting the creator's perceptions of audience engagement: (1) vicarious gratification and appetite regulation, (2) food preferences and non-vegetarian appeal, and (3) parasocial and gendered engagement dynamics. Findings suggest that, from the creator's perspective, mukbang functions as a digital site of vicarious eating, sensory stimulation, and parasocial interaction, through which viewers are perceived to regulate cravings, seek companionship, and engage with culturally embedded food practices. At the same time, the creator's account highlights tensions surrounding the normalization of excessive consumption and the “thinness paradox,” wherein high-volume eating is performed alongside maintaining physical fitness. He also highlights how online hatred fuels platform engagement. While the study does not directly examine audience behavior, it provides insight into how creators interpret and construct their audiences within platform-driven ecosystems. These findings raise important ethical and public health considerations, particularly in relation to disordered eating patterns, digital influence, and the responsibilities of content creators and platforms.

## Introduction

Mukbang, a South Korean phenomenon derived from the words meokneun (eating) and bangsong (broadcast) involves people eating and recording themselves online while impossibly gorging on a large platter of food accompanied by vivid graphic chewing sounds (Kircaburun et al., 2021a,b, Lavelle, 2018). Mukbangers film themselves consuming copious amounts of food (mostly unhealthy) while interacting with their viewers. The camera is positioned up close, so viewers face a pile of food, set against a plain backdrop to minimize distractions. They additionally present a “bite” of their food to the camera, almost seducing, goading the audience to sample their plate. A close-up sound mic amplifies the intimate sounds of chewing.

Mukbang visuals are often designed around autonomous sensory meridian response (ASMR) (Mahady et al., 2023). This is a performance geared toward creating a spectacle, for example, Nicocado Avocado's “Am I fat” video in which Nico jiggles his paunch while eating immense helpings of cheeseburgers, deep-fried nuggets, and fries slathered with melted cheese, has 22 million views on YouTube (Avocado, 2022). A video by Maddy Eats of her eating chocolate bars, and chocolate-covered eggs dipped into an impossibly large tub of molten chocolate has more than 13 million views (MaddyEats, 2023).

Originating in South Korea, the trend has meandered to many Asian countries such as Japan, China, Thailand, Vietnam, and India. Each mukbanger proudly showcases their country's delicacies and cuisine by recording themselves eating those regional specialities. In China, for instance, a trend known as “chibo” has permeated online platforms wherein content creators eat spicy foods and hot pots (Xu et al., 2022). In France, mukbangers are seen eating a wide variety of cheeses, breads, and sliced cold-cuts, including a charcuterie board (Lucile, 2023). In India, the trend has shape-shifted into food being identity, tradition, along with family (Disha, 2024). Porchezhiyan (YouTube, 2018) is a 59-year-old man who runs a channel with his son. In his videos, he eats with his hands on a banana leaf, and the ingredients are sourced from his own farm. Apei Opalic, based in Serbia, uses her platform to introduce viewers to Naga cuisine, whilst conversing with her audience (Apei, 2018).

While several studies have applied various theoretical models to investigate why individuals use online applications (Li et al., 2016; Cole and Griffiths, 2007; Demetrovics et al., 2011; Demetrovics and Király, 2016; Kardefelt-Winther, 2017), research on motives and other psychological characteristics of mukbang viewing has only come up in the recent past (Kircaburun et al., 2021a,b).

From a psychological perspective, a constellation of mechanisms involving vicarious eating, parasocial companionship, and affective soothing makes mukbang appealing to its viewers. Vicarious eating enables viewers to simulate the sensory pleasure of consuming highly palatable foods without engaging in actual intake, a process linked to cue-reactivity, reward anticipation, and appetite-related schemas (Järvinen et al., 2025). This makes mukbang especially appealing during moments of craving, late-night hunger, or restricted access to desired foods, as viewers use these videos to regulate appetite or temporarily suppress urges.

Research grounded in Uses and Gratifications Theory suggests that viewers actively seek mukbang to fulfill sensory, emotional, and social needs (Kircaburun et al., 2018), including mood modification, distraction from stress, and the comfort of witnessing unapologetic indulgence (Rubin, 2009). These gratifications are reinforced by parasocial interaction, through which repeated exposure to creators fosters feelings of intimacy and inclusion (Giles, 2002). Mukbang creators often speak directly to viewers, make prolonged eye contact with the camera, and construct a sense of co-presence, a digital equivalent of “eating together” (Lee, 2004). For many, this serves as social surrogacy, alleviating loneliness or unmet relational needs (Derrick et al., 2009).

Collectively, these interlinked mechanisms, sensory immersion, emotional regulation, and parasocial bonding offer a psychologically grounded explanation for the persistent appeal and immersive nature of mukbang consumption.

## Motivations and spectrums to mukbang watching and creation

### Loneliness

The primary role of mukbangs is “social facilitation”. Multiple studies highlight that viewers seek a sense of *co-presence* and *companionship* through these videos, especially when living alone or experiencing social isolation (Song, 2018; Anjani et al., 2020; Kircaburun et al., 2021a,b). The interactive features, such as live comments and responses from the host, enhance feelings of community and reduce lonesomeness (Kircaburun et al., 2021a,b). The act of “digital commensality” (Kircaburun et al., 2018), the act of eating together, creates a virtual community, and therefore supposedly reduces loneliness for both the creator and the viewer. Hakimey and Yazdanifard (2015) argued that “emotional connection” along with feelings of “empathy” toward the mukbanger are crucial factors contributing to viewership.

### Escapism

Mukbangs have also become an escape route, a sort of instant relief. It gives viewers a stimulating sense of indulging in decadent cuisine through vicarious, virtual eating (Kircaburun et al., 2020). Bruno and Chung (2017) claim that viewers watch mukbangs to escape feelings of stress and guilt about being fat. Mukbangs have additionally developed into a form of vicarious delight for children and teenagers, whose parents have forbidden them from ordering food outside (Bruno and Chung, 2017).

### Voyeurism

Food sexualization and mediated voyeurism are less frequently discussed but remain important factors in viewer engagement. Some mukbang videos openly or inadvertently sexualize eating in an attempt to entice viewers who engage in voyeuristic or sexual enjoyment from the material (Bruno and Chung, 2017; Kircaburun et al., 2021a,b). Research indicates that the act of watching someone eat in an intimate, unguarded manner can be inherently voyeuristic, and, in some cases, the sensual presentation of eating may blur boundaries between food and sexuality (Bruno and Chung, 2017). A study in 2018 revealed that women mukbangers are “sexually objectified”, and possibly “fetishized” (Schwegler-Castañer, 2018).

Gillespie (2020) analyzed a total of 36 videos consisting of nine female mukbangers across countries such as the USA, Canada and South Korea. The study found that viewers derived pleasure from watching women eat large portions, in a messy manner, and loudly. Research points to the fetishization and sexual objectification of female mukbangers amplifying harmful societal standards regarding body image and desirability (Sanskriti et al., 2023).

A few mukbangers encourage toxic viewer engagement through provocative content, thus reflecting how social media algorithms prioritize digital interaction (irrespective of whether positive or negative) and disregard the content's implications.

Viewers' comments can be sexually explicit, as in the prominent female mukbanger Janie's YouTube channel (Janie, 2022), where one can find comments such as, “*Okay…. But you‘re doing a lot with the sausage lol”, and “Her man must be happy,”* (Janie, 2022).

Women portray themselves as having large and “shameful” appetites. This creates a vulnerable and intimate setting, which in turn “sexualises physical characteristics of women” (Sanskriti et al., 2023). The study pointed out that “sociability” and “general likeability” among women also drew viewers (Sanskriti et al., 2023). Bijolia (2017) revealed that women mukbangers who were considered conventionally attractive and thin primarily had overweight men as their audience, for young adults who may be vulnerable, this can lead to threats, as it may influence their body standards.

### Autonomous sensory meridian response (ASMR)

ASMR is a major draw for many mukbang viewers. The distinct sounds of eating ie. chewing, slurping, and crunching can trigger ASMR, which is associated with pleasurable tingling sensations and relaxation (Kircaburun et al., 2021a,b). Studies show that ASMR mukbang videos are especially popular for stress relief, relaxation, and even as a sleep aid (Jiang et al., 2024), the immersive audio-visual experience not only enhances viewers' perceptions of taste and food enjoyment but also provides vicarious sensory satisfaction (Kircaburun et al., 2021a,b).

### Problematic eating behaviors

Mukbang allows for vicarious eating, satisfying cravings or dietary restrictions without actual consumption (Kircaburun et al., 2021a,b; Choe, 2019). Some viewers use mukbang to manage their own eating behaviors, either by curbing appetite or providing psychological satiation (Kircaburun et al., 2021a,b). However, concerns have been raised regarding the normalization of overeating and unhealthy eating patterns, as mukbang often features excessive food consumption (Kircaburun et al., 2021a,b; Anjani et al., 2020). Research also points to the potential for mukbang to influence viewers' real-life food choices and purchasing behaviors, especially among younger audiences (Song, 2018; Kircaburun et al., 2021a,b). Recent studies also show that higher mukbang watching frequency is significantly associated with external eating behaviors, such as eating in response to external cues rather than internal hunger signals. This may increase susceptibility to overeating and obesity (Kircaburun et al., 2021a,b; Anari and Shahryar, 2023).

## The growth of mukbang in India

Over the past few years, mukbang has seen a remarkable surge in Indian online searches and YouTube content. According to Google Trends data, the term “mukbang” became three times more popular in India between 2018 and 2019, reflecting a sharp increase in both content creation and viewership (Anjali, 2019). Indian mukbangers such as “The Indian Mukbanger” (Das, 2021) have amassed more than 3 million subscribers (Disha, 2024). Moreover, Venugopalan (2019) highlights that many creators are motivated not only by financial opportunities (mukbang usually earn between $150 and $3,000 a month through advertising and brand promotions), but also by the social rewards of building loyal communities and directly engaging with their audiences.

Complementing this perspective, the genre's appeal in India is enhanced by its integration with regional food traditions, vernacular languages, and the blending of global and indigenous eating practices. However, the pursuit of virality and sustained audience engagement can expose creators to body shaming, online harassment, and mental health challenges, as seen in the case of the popular content creator Janie's comment section in a YouTube video (Janie, 2022).

While prior research has predominantly focused on viewer motivations, psychological engagement, and patterns of consumption in mukbang, comparatively little attention has been paid to how content creators themselves interpret audience behavior and shape their production practices.

## Methods

### Study design

To examine how mukbang engagement is understood and interpreted within the Indian digital context, this study employed an interpretive qualitative single-case study design. Single-case designs are well-established in exploratory and interpretive research, particularly when investigating information-rich or hard-to-access phenomena (Flyvbjerg, 2006; Yin, 2018; Stake, 1995). A case study approach was considered appropriate given the relative lack of empirical research on mukbang in India, as it enables in-depth, contextually grounded exploration of complex and under-researched digital practices.

The study focused on a purposefully selected, information-rich case: a prominent Indian mukbang and fitness content creator. The participant “A” was selected due to his substantial digital presence and dual role as both content producer and interpreter of audience engagement, enabling access to experiential and platform-specific insights that would be difficult to obtain through broader sampling strategies. Rather than generating direct evidence of audience behavior, this design facilitates examination of the participant's situated interpretations of audience engagement alongside his own content production practices.

The study adopted a theory-informed interpretive approach. The semi-structured interview was guided by prior empirical literature on mukbang and digital eating behaviors, including constructs such as vicarious gratification, parasocial interaction, and gendered engagement. These constructs functioned as sensitizing concepts to explore how the participant made sense of his audience and content ecosystem, rather than as hypotheses to be tested or validated.

Accordingly, the study is best understood as interpretive and theory-guided, aiming to examine how broader patterns identified in the literature are perceived, articulated, and contextually adapted by a creator operating within the Indian digital landscape. The findings are not intended to be generalizable to wider populations of creators or viewers; instead, they offer a situated, context-specific account that contributes to theoretical and conceptual understanding, and may inform future research incorporating multiple stakeholders and empirical audience data.

### Participant and recruitment

A prominent 29-year-old male Indian mukbanger and fitness content creator (“A”) was recruited through direct contact via his email available on YouTube. The participant was purposively selected as an information-rich case due to his substantial digital presence, dual role as both mukbanger and fitness content creator, and professionalized content production practices, which enabled insight into both content creation and perceived audience engagement.

Participation was voluntary, and no incentives were provided. Ethical approval for the study was obtained from the Institutional Review Board (IRB), FLAME University, Pune, India (Approval No. 2025/04/06/UG/EXP), under expedited review procedures, and the study was deemed to involve minimal risk to the participant. All procedures were conducted in accordance with institutional ethical guidelines and the Declaration of Helsinki (World Medical Association, 2024).

The participant provided informed consent prior to participation, including consent for audio and video recording and the use of anonymised data in publication. Given the participant's public-facing role, complete anonymisation cannot be fully guaranteed; this limitation was explicitly discussed during the consent process, and identifying details have been minimized where possible.

### Data collection

The semi-structured interview, which was guided by earlier empirical findings, went beyond open exploration to look at audience dynamics, psychological aspects, and known motivations as perceived and expressed by the content creator and explored motivations for producing mukbang videos, perceptions of audience engagement, fetishization around food, views on cravings, ASMR, gendered patterns of consumption, and perceived psychological impacts on viewers. The 75-min interview was audio-recorded and transcribed.

### Analytic approach

Data were analyzed using Braun and Clarke's (2006) reflexive thematic analysis. The analysis proceeded through six iterative phases: (1) familiarization with the transcript, (2) initial coding using a semantic–latent approach, (3) generation of candidate themes, (4) reviewing and refining themes, (5) defining and naming themes, and (6) producing the final narrative account. Coding and theme development were conducted manually to allow close interpretive engagement with the data. A reflexive approach was adopted throughout the research process to account for the interpretive nature of qualitative analysis. The participant in this study is a high-visibility content creator, whose responses may reflect a practiced and publicly mediated narrative about his work and audience. This introduces the possibility that the data represent a curated self-presentation shaped by platform norms and audience expectations.

In addition, the researchers brought prior familiarity with the mukbang literature, particularly in relation to parasocial interaction, vicarious consumption, and digital eating behaviors. These theoretical orientations informed both the design of the interview guide and the interpretation of the data, potentially shaping the identification and prioritization of themes.

No member-checking was conducted, and the participant was not involved in reviewing or validating the interpretations presented. As such, the findings represent a researcher-led interpretation of the participant's account rather than a co-constructed or participant-verified narrative. These considerations were actively reflected upon during analysis to mitigate overinterpretation and ensure alignment between the data and the claims made.

### Case study of “A”

To better understand the cultural adaptation of mukbang in India, we conducted an in-depth qualitative interview with Mr. “A,” a 29-year-old prominent Indian mukbanger and fitness content creator with close to one million YouTube subscribers. He currently resides in Uttarakhand, India. The interview was conducted via Zoom and sought to explore broader questions such as: *What attracts audiences to mukbangs? What socio-cultural factors contributed to the rise of this trend in India? What are the psychological implications for both creators and consumers?*

### Background and entry into content creation

“A” is a 29-year-old male, Indian content creator based in Uttarakhand, northern India, with close to one million YouTube subscribers across separate mukbang and fitness channels. Prior to content creation, he worked in the media industry, an experience that equipped him with technical filmmaking skills directly applicable to YouTube production. His entry into mukbang emerged organically from an existing fitness content practice, through which he identified an opportunity to integrate indulgent eating with health-conscious messaging - a combination he attributes to his subsequent audience growth. This dual positioning, as both a mukbang creator and a fitness figure, is analytically significant as it embodies the “thinness paradox” that structures much of his content and audience engagement.

### Motivations and audience engagement

When asked why audiences consume mukbang content, “A” emphasized vicarious gratification: “*Logon ki aadhi craving khatam ho jaati hai jab wo kisi ko khate hue dekhte hain”* (people's cravings are half satisfied when they watch someone else eating). He highlighted that mukbangs are particularly popular late in the night, when food cravings are heightened but access to food is limited.

In addition to suppressing cravings, mukbangs also address social isolation. According to “A”: “*Jo baahar log akele reh rahe hain wo khud ko itna akela feel na karein”* (people living alone do not feel as lonely when someone interacts with them while eating).

### Audience demographics

“A” observed that mukbang videos that contain meat consistently attract more views than vegetarian ones, aligning with broader food preferences in India. He reported his analytics showed that the majority of his audience (approximately 65%) falls between the ages of 18–24, followed by viewers aged 24–35. These audience metrics were reported by the participant and were not independently verified.

Regarding gender dynamics, he suggested that women mukbangers generally attract larger audiences. This, he argued, is partly because female viewers relate to their audiences, while male viewers may be drawn to both the content and to the creators themselves. He also reported that, in his view, men tend to have more alternative leisure activities, whereas women are “more specific about satisfying their moods” through such content.

### The rise of mukbang in India

Reflecting on mukbang's global diffusion, “A” reported that the trend originated in South Korea between 2008 and 2010 as a response to urban loneliness. In India, its appeal lies in its simplicity: “*Ye trend isliye aaya kyunki it was the easiest thing…basically aapko khana khana hai”* (this trend came because it was the easiest—essentially, one only has to eat on camera). He sees mukbangs as an accessible entry point for aspiring creators in India's rapidly growing digital ecosystem.

### Health implications and mixed messages

The discussion also explored potential risks. “A” acknowledged that mukbangs can convey mixed messages, especially to impressionable young audiences. While some viewers use mukbangs to control late-night cravings and avoid overeating, others are inspired to emulate excessive eating, potentially fostering binge eating or disordered eating behaviors.

He recounted a tragic instance of an Indian mukbanger's death from complications linked to excessive food consumption, underscoring the dangers. To mitigate risks, “A” emphasized the responsibility of creators to balance indulgence with fitness messaging: “*Enjoy food, but dhyaan do”* (enjoy the food you eat, but pay attention to your health).

### Creator strategies for fitness and sustainability

To manage the physical consequences of mukbang filming, “A” follows a One Meal a Day (OMAD) regimen (Malavika, 2026), a regimen he concedes is “not sustainable” but effective for him. On high-calorie filming days, he fasts for a full day afterward, and in cases of pre-shooting, he undertakes water fasts of up to 72 h. He works with a dietitian and a personal trainer to maintain his health.

### The future of mukbang in India

Regarding sustainability, “A” predicted that while current trends favor short-form content (e.g., YouTube Shorts, Instagram Reels), long-form videos will regain prominence as they offer stronger branding and revenue opportunities. He sees mukbangs as having significant growth potential in India once this shift occurs.

### Monetization and platform dynamics

On monetization, “A” explained that creators must reach 1,000 subscribers and 4,000 watch hours to qualify for YouTube's Partner Program (Google.com, 2025). He noted that beyond views, engagement through comments is particularly crucial, as it signals algorithmic relevance: “*Aaj ki date mein jo bhi YouTube mein ya kahin bhi social media se paise kama raha hai, usse zyada dimaag wala insaan koi nahin hai”* (in today's world, anyone earning money from YouTube or social media is among the smartest).

## Results

Using Braun and Clarke's (2006) six-phase reflexive thematic analysis, one overarching theme was developed: Audience Engagement and Content Dynamics, with three subthemes: (1) Vicarious Gratification and Appetite Regulation, (2) Food Preferences and Non-Vegetarian Appeal, and (3) Parasocial and Gendered Engagement Dynamics. These subthemes capture how “A” perceives audience motivations, consumption patterns, and demographic differences in engagement with mukbang content.

### Vicarious gratification and appetite regulation

A recurring idea was that mukbangs were described by the participant as functioning as a substitute for direct food consumption, particularly late at night. “A” described how viewers may use his videos to manage cravings in contexts where food is not easily available: “*Raat ko itne logon pe option nahin rehte hain. But craving poori rehti hai”* (At night, people don't have many options, but cravings persist). Watching others eat was described by the participant as providing vicarious satisfaction: “*Logon ki aadhi craving khatam ho jaati hai jab wo kisi ko khate hue dekhte hain”* (People's cravings are half satisfied when they see someone else eating). For some viewers, this effect diminished their desire to eat altogether: “*Apni ichchha mar gayi ki yaar theek hai yaar bas ho gaya. Sote hain ab”* (Their desire dies down, and they think, that's enough, let's sleep now). These accounts illustrate how the participant conceptualized mukbang as operating as a form of parasocial regulation of appetite and cravings.

### Food preferences and non-vegetarian appeal

“A” also emphasized the centrality of food type in shaping audience engagement. He reported observing a consistent preference for non-vegetarian content: “*The ratio of non-vegetarians is higher*.” This was interpreted by the participant as suggesting that sensory appeal and cultural associations with indulgence may amplify the attractiveness of non-vegetarian foods, positioning them as a dominant driver of viewership within his channel.

### Parasocial and gendered engagement dynamics

The participant further identified gendered dynamics in audience behavior. He suggested that men and women may engage with mukbangs differently: “*Ladkon ke paas time waste karne ke liye bahut cheezen hain*” (men have many things to waste their time on), whereas women are “more specific about satisfying their moods,” making them more likely to engage with such content. Additionally, he indicated that female mukbang creators may attract a dual audience, appealing to women through relatability and to men through physical attractiveness: “*Main mukbangers ko ladkiyan to dekhti hi hain, saath mein ladke bhi dekhte hain ki haan ye achhi lag rahi hai*” (Female audiences watch mukbang, but men also watch because they find women mukbangers appealing). This was described by the participant as reflecting gendered asymmetries in how parasocial interest intersects with entertainment, consumption, gender and attraction.

In addition to capturing audience dynamics, these themes also provide insight into the participant's approach to content creation. “A's” interpretations of viewer cravings, food preferences, and gendered engagement suggest that his content decisions may be informed by ongoing audience monitoring and adaptive learning. His emphasis on late-night cravings, non-vegetarian appeal, and differential gender engagement indicates a form of intuitive audience modeling, in which he appears to anticipate and respond to perceived viewer needs and preferences.

From the participant's perspective, this reflects a feedback-driven creative process shaped by reinforcement contingencies such as views, comments, and audience retention, through which content is iteratively adjusted to sustain engagement. At the same time, his awareness of gendered attraction and relatability highlights the role of self-presentation and performative identity in mukbang production, suggesting that he actively negotiates how he appears, what he consumes, and how he engages with viewers within platform-specific norms and expectations.

## Discussion

This case study examined how mukbang is interpreted in the Indian context, highlighting the intersections among appetite regulation, affective coping, sensory engagement, and parasocial interaction. The participant described mukbang functioning not only as entertainment but also as a psychological tool through which viewers manage cravings, negotiate loneliness, and engage with digital intimacy. These themes resonate with global research positioning mukbang as a cultural practice with implications for health, wellbeing, and online behavior (Kircaburun et al., 2021a,b). It is important to note that these interpretations reflect the participant's perceptions of audience engagement rather than direct empirical evidence of viewer behavior, and should be understood within the limits of a single-case, interpretive design.

### Vicarious gratification and affective regulation

A central finding concerns the use of mukbang for vicarious gratification, especially during late-night viewing when cravings are heightened. “A” described viewers “eating through others,” an observation consistent with work showing that virtual food consumption can provide partial satiety and temporary craving relief (Kircaburun et al., 2021a,b). These dynamics align with theories of cue-reactivity and affect regulation, which propose that appetitive cues may either suppress or intensify cravings depending on an individual's motivational state (Boswell and Kober, 2015; Gross, 1998).

However, this relief appears to be dual in nature. While some of “A's” viewers report reduced desire to consume food after watching mukbangs, others may experience heightened sensitivity to food cues, which could increase the likelihood of subsequent overeating. Such patterns have been reported in controlled studies where visual exposure to palatable foods both satisfies and stimulates desire (Hong and Park, 2017). These findings highlight the need to contextualize mukbang within broader psychological processes of modeling, reinforcement, and reward anticipation.

### Parasocial interaction and digital companionship

The participant's reflections emphasized the role of mukbang in alleviating feelings of isolation. Viewers reportedly rely on mukbang videos to simulate shared mealtimes, which aligns with established research on parasocial interaction and media-based social surrogacy (Horton and Wohl, 1956; Derrick et al., 2009). For young adults living alone or away from family, mukbang appears to offer a sense of co-presence and routine. Comment sections also function as communal spaces that foster belonging, connection, and shared emotional expression. This raises important questions about whether digital companionship complements or displaces offline social interaction—an area requiring further empirical examination.

### Sensory reinforcement and food preferences

Food type emerged as a significant driver of engagement. Consistent with the participant's analytics, non-vegetarian mukbangs attracted higher viewership than vegetarian content. This pattern likely reflects the sensory richness and perceived indulgence associated with meat dishes, which may amplify reward-based engagement. Similar trends have been documented in East and Southeast Asian mukbang cultures, where visually intense, high-calorie foods dominate (Choe, 2019). Moreover, ASMR components commonly embedded in mukbang may enhance viewer immersion by eliciting physiological calming and sensory pleasure (Poerio et al., 2018).

### Gendered patterns of engagement

Gendered differences in viewing behavior were also evident. The participant reported that women engage more intentionally with mukbangs, whereas men may be more sporadic consumers. Female creators reportedly attract both women (via relatability) and men (via perceived attractiveness), echoing work on gendered parasocial relationships and visibility in digital culture (Sanskriti et al., 2023). These findings underscore the gendered labor embedded in digital eating performances and the asymmetrical expectations placed on women creators.

### Body image concerns and the paradox of thinness

A notable concern arising from the interview relates to the “thinness paradox,” wherein creators maintain slim or muscular physiques despite displaying extreme consumption. Prior research suggests that mukbang can idealize a fantasy of consequence-free eating, potentially contributing to distorted beliefs about weight regulation and bodily norms (Hong and Park, 2017). For adolescents and young adults, such portrayals may exacerbate body dissatisfaction or encourage unhealthy eating behaviors. These issues also relate to broader discussions about the invisibility of bodily labor, fasting, exercise, or editing behind seemingly effortless performances.

The participant's reported practices of consuming large quantities of food during filming followed by prolonged fasting, including water-only fasts of up to 72 h, and habitual restriction through one-meal-a-day patterns bear resemblance to compensatory behaviors described in the clinical literature on disordered eating. Such patterns are consistent with binge–restrict cycles documented in both clinical and subclinical populations (Flayelle et al., 2020), and share features with disorders characterized by recurrent episodes of excessive intake followed by compensatory strategies aimed at weight control (e.g., American Psychiatric Association, 2013; World Health Organization, 2019).

While this study does not involve clinical assessment and does not seek to diagnose the participant, these behaviors raise important concerns regarding the normalization of compensatory eating patterns within digitally mediated food performances. The participant's acknowledgment that these practices are “not sustainable” further reflects an awareness of their potential harm, aligning with literature that highlights the psychological and physiological risks associated with such cycles.

These observations also intersect with emerging research on how platform incentives, monetization structures, and audience engagement may reinforce extreme or unsustainable eating behaviors among content creators. In this sense, mukbang production may not only reflect but also potentially sustain patterns that warrant closer examination within clinical and public health frameworks.

### Health risks and platform responsibility

The dietary concerns raised by “A” represent significant public health concerns that extend beyond the interests of the creator. To compensate for the caloric excess associated with mukbang filming, “A” follows an OMAD protocol and fasts for up to 72 h under the guidance of a licensed nutritionist. While intermittent fasting has received clinical attention, extreme variants such as OMAD remain poorly evidenced, and prolonged fasting has been associated with muscle catabolism, micronutrient deficiency, and heightened risk of binge-restrict cycles (Cioffi et al., 2018; Harris et al., 2018). These patterns of large-scale eating followed by compensatory restriction potentially mirror behaviors associated with binge eating disorder and purging disorder as classified in the DSM-5 (American Psychiatric Association, 2013).

The more pressing concern here, however, is the asymmetrical power relationship between creator and viewer. Professional mukbangers frequently work alongside nutritionists and personal trainers to manage the physical consequences of their content (Strand and Gustafsson, 2020). Young viewers who may imitate what they observe—consuming large quantities of food and normalizing restrict-binge patterns—do so without this professional scaffolding and without awareness that mukbang is a heavily managed performance rather than a sustainable lifestyle (Kircaburun et al., 2021a,b). This is particularly concerning given that “A” reports 65% of his audience falls within the 18–24 age bracket, a demographic known to be vulnerable to disordered eating and susceptible to social media influence on food attitudes (Rodgers et al., 2020). This study therefore contributes to calls for greater creator transparency and platform-level health considerations around extreme eating content.

The health risks associated with mukbang are not merely hypothetical. Recent tragedies underscore their severity: a Filipino mukbanger reportedly died of complications linked to overeating (The Philippine Daily Inquirer/Asia News Network, 2024), and Chinese livestreamer Pan Xiaoting, aged 24, died after a prolonged binge-eating session (TOI World Desk, 2024). These cases highlight the very real dangers of incentivising excessive consumption as digital performance. According to YouTube, (YouTube, 2019) as part of their content policies, YouTube explicitly prohibit “harmful or dangerous acts,” yet enforcement appears inconsistent.

The persistence of mukbang content raises questions about algorithmic responsibility, monetization structures, and the ethics of promoting high-risk behaviors under the guise of entertainment.

### Policy and ethical implications

Despite YouTube's Community Guidelines prohibiting content that endangers health and safety (YouTube, 2019), no platform-level statutory warnings exist specifically for mukbang content depicting extreme eating behaviors. Unlike tobacco and alcohol, where health warnings are legally mandated across media (World Health Organization, 2025), mukbang videos normalizing binge eating and compensatory restriction remain unregulated at the content level (Kircaburun et al., 2021a,b). This study recommends the introduction of viewer-facing health disclaimers before such content, alongside broader regulatory frameworks that hold digital platforms accountable for amplifying consumption behaviors with demonstrable public health consequences.

According to Covington and colleagues (2016), YouTube primarily maintains viewer engagement through a thorough two-stage process (deep neural network system). The first stage involves ca**n**didate generation, selecting a relevant subset of videos from millions based on user history and context. The second stage, ranking, scores these candidates using hundreds of specialized features. Crucially, the system ranks videos by “expected watch time” rather than simple click-through rates, which prevents “clickbait” and ensures users find content satisfying enough to complete. Additionally, the algorithm incorporates “freshness” signals to promote new content and adjusts recommendations based on recent user impressions.

YouTube's recommendation system usually prioritizes expected watch time over simple clicks, which makes long-form, sensory-driven content like mukbang particularly compatible with algorithmic promotion (Covington et al., 2016).

Given these concerns, one recommendation is the introduction of statutory warnings before mukbang videos, akin to health disclaimers on tobacco and alcohol. Such warnings could inform viewers about the risks of binge eating and reinforce that the behaviors depicted are not without health consequences. More broadly, there is an urgent need for regulatory frameworks that consider not only individual responsibility but also the role of digital platforms in shaping consumption practices and health outcomes.

### Content creation as a psychological process

The findings also point to the importance of conceptualizing mukbang not only as a phenomenon of video content consumption, but also as a psychologically complex process of content creation shaped by platform dynamics and audience feedback. From the participant's perspective, mukbang production appears to involve processes of strategic self-presentation, reinforcement learning, and ongoing identity negotiation.

Decisions regarding food type, portion size, and performance style were described as being influenced by platform analytics, audience engagement patterns, and monetization incentives, suggesting the presence of a feedback loop in which certain behaviors such as exaggerated consumption or visually stimulating food displays may be selectively reinforced. At the same time, the participant highlighted the need to balance authenticity with performativity, while navigating pressures related to body image, visibility, and gendered expectations.

These dynamics position mukbang creation as a form of affective and embodied digital labor, in which psychological regulation extends beyond viewers to include creators, who continuously adapt their behavior in response to both internal motivations and external platform-driven contingencies.

## Limitations and future directions

This study is based on a single, information-rich case and reflects a situated account of one content creator's interpretations of audience engagement and content production practices. As such, the findings are not intended to be generalizable to broader populations of creators or viewers. Rather, they provide a context-specific, interpretive insight into how mukbang is understood and enacted within a particular digital context.

Importantly, the analysis relies on the participant's self-reported perceptions of audience behavior and platform analytics, which were not independently verified. Consequently, the findings should not be interpreted as direct evidence of viewer motivations or psychological processes, but as a creator's perspective on these dynamics.

In addition, while the study engages with clinical frameworks to interpret reported eating practices, it does not involve clinical assessment and cannot establish the presence of disordered eating. Future research should therefore incorporate multi-perspective designs, including direct data from viewers, creators across varying scales of visibility, and where appropriate, clinically informed assessments of eating behaviors.

Further work may also benefit from integrating platform-level data and longitudinal approaches to examine how digital content production, audience engagement, and eating behaviors interact over time. Such approaches would help clarify the psychological and health implications of mukbang within increasingly monetised and algorithm-driven digital environments.

## Conclusion

Mukbang represents a cultural and psychological paradox: it simultaneously satisfies and stimulates cravings, fosters connection while promoting isolation, and celebrates indulgence while concealing its costs. This case study adds to a growing body of work that situates mukbang within cyberpsychology as a site where digital culture, embodiment, and health intersect. Future research must continue to explore how such practices are localized in non-Korean contexts, how they shape youth relationships with food and body image, and how digital platforms can be held accountable for the health implications of monetized eating.

## Data Availability

The datasets presented in this article are not readily available because they contain a video interview of a famous mukbanger. The content creator has not given consent to release their identity. Requests to access the datasets should be directed to gayatri.kotbagi@flame.edu.in.

## References

[B1] American Psychiatric Association (2013). “Diagnostic and statistical manual of mental disorders,” in Diagnostic and Statistical Manual of Mental Disorders, 5th ed. (Arlington, VA: American Psychiatric Association). doi: 10.1176/appi.books.9780890425596

[B2] Anari F. M. Shahryar E. (2023). The relationship between watching mukbang (eating show), eating behaviors, and anthropometric parameters in iranian female students. J. Res. Health Sci. 23:e00574. doi: 10.34172/jrhs.2023.109 37571945 PMC10422146

[B3] Anjali V. (2019). Feast & Stream: Meet India's Biggest Mukbangers. Mumbai: The Economic Times. Available online at: https://economictimes.indiatimes.com/magazines/panache/feast-stream-meet-indias-biggest-mukbangers/articleshow/71027715.cms?from=mdr (Accessed May 26, 2026).

[B4] Anjani L. Mok T. Tang A. Oehlberg L. Goh W. B. (2020). “Why do people watch others eat food? An empirical study on the motivations and practices of Mukbang viewers,” in Proceedings of the 2020 CHI Conference on Human Factors in Computing Systems (Calgary, AI: Association for Computing Machinery), 1–13. doi: 10.1145/3313831.3376567

[B5] Apei (2018). Apei Eats. Belgrade: YouTube. Available online at: https://www.youtube.com/channel/UC-2qCIkUSlrVUVmMy8J1MRA (Accessed October 8, 2025).

[B6] Avocado (2022). Nicocadoshorts. Am I Fat...? #shorts. Available online at: https://www.youtube.com/shorts/A5pnuPKWB9Q (Accessed October 1, 2025).

[B7] Bijolia D. (2017). ‘Food porn' or intimate sociality: committed celebrity and cultural performances of overeating in meokbang. Celebrity Stud. 8, 122–127. doi: 10.1080/19392397.2016.127285721539050

[B8] Boswell R. G. Kober H. (2015). Food cue reactivity and craving predict eating and weight gain: a meta-analytic review. Obes. Rev. 17, 159–177. doi: 10.1111/obr.12354 26644270 PMC6042864

[B9] Braun V. Clarke V. (2006). Using thematic analysis in psychology. Qual. Res. Psychol. 3, 77–101. doi: 10.1191/1478088706qp063oa

[B10] Bruno A. L. Chung S. (2017). Mokpang: pay me and I'll show you how much I can eat for your pleasure. J. Jap. Korean Cinema 9, 155–171. doi: 10.1080/17564905.2017.1368150

[B11] Choe H. (2019). Eating together multimodally: collaborative eating in mukbang, a Korean livestream of eating. Lang. Soc. 48, 171–208. doi: 10.1017/S0047404518001355

[B12] Cioffi I. Evangelista A. Ponzo V. Ciccone G. Soldati L. Santarpia L. . (2018). Intermittent versus continuous energy restriction on weight loss and cardiometabolic outcomes: a systematic review and meta-analysis of randomized controlled trials. J. Transl. Med. 16:371. doi: 10.1186/s12967-018-1748-4 30583725 PMC6304782

[B13] Cole H. Griffiths M. D. (2007). Social interactions in massively multiplayer online role-playing gamers. Cyberpsychol. Behav. 10, 575–583. doi: 10.1089/cpb.2007.9988 17711367

[B14] Covington P. Adams J. Sargin E. (2016). “Deep neural networks for youtube recommendations,” in Proceedings of the 10th ACM Conference on Recommender Systems - RecSys '16 (New York, NY: Association for Computing Machinery), 191–198. doi: 10.1145/2959100.2959190

[B15] Das S. (2021). The Indian Mukbanger. YouTube. Available online at: https://www.youtube.com/channel/UCXzOPXsOLCJQcy1LUtUaUdg (Accessed May 27, 2026).

[B16] Demetrovics Z. Király O. (2016). Commentary on Baggio et al. (2016): internet/gaming addiction is more than heavy use over time. Addiction 111, 523–524. doi: 10.1111/add.13244 26860248

[B17] Demetrovics Z. Urbán R. Nagygyörgy K. Farkas J. Zilahy D. Mervó B. . (2011). Why do you play? The development of the motives for online gaming questionnaire (MOGQ). Behav. Res. Methods 43, 814–825. doi: 10.3758/s13428-011-0091-y 21487899

[B18] Derrick J. L. Gabriel S. Hugenberg K. (2009). Social surrogacy: how favored television programs provide the experience of belonging. J. Exp. Soc. Psychol. 45, 352–362. doi: 10.1016/j.jesp.2008.12.003

[B19] Disha B. (2024). Eating Alone; Together: How Indian ‘Mukbangers' are Changing Food Culture Online. Mumbai: Homegrown. Available online at: https://homegrown.co.in/homegrown-explore/eating-alone-together-how-indian-mukbangers-are-changing-food-culture-online (Accessed May 26, 2026).

[B20] Flayelle M. Maurage P. Lorenzo D. i. Vögele K. R. Gainsbury C. Billieux S. M. . (2020). Binge-watching: what do we know so far? A first systematic review of the evidence. Curr. Addict. Rep. 7, 44–60. doi: 10.1007/s40429-020-00299-8

[B21] Flyvbjerg B. (2006). Five misunderstandings about case-study research. Qual. Inq. 12, 219–245. doi: 10.1177/1077800405284363

[B22] Giles D. C. (2002). Parasocial interaction: a review of the literature and a model for future research. Media Psychol. 4, 279–305. doi: 10.1207/S1532785XMEP0403_04

[B23] Gillespie S. (2020). Watching women eat: a critique of magical eating and mukbang videos. Available online at: https://www.proquest.com/docview/2307190763/EF41087BAE4A4C9DPQ/4?fromunauthdoc=true&sourcetype=Dissertations%20&%20Theses (Accessed May 6, 2026).

[B24] Google.com. (2025). YouTube Partner Program – YouTube channel monetization policies. [online]. Available online at: https://support.google.com/youtube/answer/1311392?hl=en (Accessed May 27, 2026).

[B25] Gross J. J. (1998). The emerging field of emotion regulation: an integrative review. Rev. Gen. Psychol. 2, 271–299. doi: 10.1037/1089-2680.2.3.271

[B26] Hakimey H. Yazdanifard R. (2015). The review of mokbang (broadcast eating) phenomena and its relations with South Korean culture and society. Int. J. Manage. Accounting Econ. 2, 444–456. doi: 10.5281/zenodo.17284454

[B27] Harris L. Hamilton S. Azevedo L. B. Olajide J. de Brún C. Waller G. . (2018). Intermittent fasting interventions for treatment of overweight and obesity in adults. JBI Database Syst. Rev. Implementation Rep. 16, 507–547. doi: 10.11124/JBISRIR-2016-003248 29419624

[B28] Hong S. Park S. (2017). “Internet Mukbang (foodcasting) in South Korea,” in Young & Creative: Digital Technologies Empowering Children in Everyday Life, eds. I. Eleá and L. Mikos (Gothenburg, Sweden: Nordicom).

[B29] Horton D. Wohl R. (1956). Mass communication and para-social interaction: observations on intimacy at a distance. Psychiatry 19, 215–229. doi: 10.1080/00332747.1956.11023049 13359569

[B30] Janie (2022). ASMR CREAMY CHEESY ALFREDO + *KIELBASA SAUSAGE MUKBANG | Eating Sounds #bigbites #creamyalfredo*. YouTube. Available online at: https://www.youtube.com/watch?v=EO8s1CHEGNc (Accessed May 27, 2026).

[B31] Järvinen L. Santavirta S. Putkinen V. Karlsson H. K. Seppälä K. Sun L. . (2025). Reward responses to vicarious feeding depend on body mass index. Cognit. Affective Behav. Neurosci. 25, 607–617. doi: 10.3758/s13415-025-01265-5 39966303 PMC12130106

[B32] Jiang N. Khong K. W. Chen M. Khoo K. L. Xavier J. N. Jambulingam M. (2024). Why am I obsessed with viewing mukbang ASMR? The roles of mediated voyeurism and intertemporal choice. PLoS ONE 19:e0308549. doi: 10.1371/journal.pone.0308549 39298369 PMC11412535

[B33] Kardefelt-Winther D. (2017). Conceptualizing internet use disorders: addiction or coping process? Psychiatry Clin. Neurosci. 71, 459–466. doi: 10.1111/pcn.12413 27278653

[B34] Kircaburun K. Alhabash S. Tosuntaş S. B. Griffiths M. D. (2018). Uses and gratifications of problematic social media use among university students: a simultaneous examination of the big five of personality traits, social media platforms, and social media use motives. Int. J. Mental Health Addict. 18, 525–547. doi: 10.1007/s11469-018-9940-6

[B35] Kircaburun K. Harris A. Calado F. Griffiths M. D. (2021a). The psychology of mukbang watching: a scoping review of the academic and non-academic literature. Int. J. Mental Health Addict. 19, 1190–1213. doi: 10.1007/s11469-019-00211-0

[B36] Kircaburun K. Savcı M. Emirtekin E. Griffiths M. D. (2020). The role of perceived feelings of presence and escapism in problematic mukbang watching among emerging adult mukbang watchers. *J. Concurr. Dis*. 2, 16–22.

[B37] Kircaburun K. Stavropoulos V. Harris A. Calado F. Emirtekin E. Griffiths M. D. . (2021b). Development and validation of the Mukbang addiction scale. Int. J. Mental Health Addict. 19, 1031–1044. doi: 10.1007/s11469-019-00210-1

[B38] Lavelle D. (2018). Mukbang: Is Loneliness Behind the Craze for Watching Other People Eating? The Guardian.

[B39] Lee K. M. (2004). Presence, explicated. Commun. Theory 14, 27–50. doi: 10.1111/j.1468-2885.2004.tb00302.x

[B40] Li J. Galley M. Brockett C. Spithourakis G. Gao J. Dolan B. . (2016). A persona-based neural conversation model. Assoc. Comput. Ling. 1, 994–1003. doi: 10.18653/v1/P16-1094

[B41] Lucile (2023). French food tour in Paris, France (by a Local)! [online] YouTube. Available online at: https://www.youtube.com/watch?v=nrDogPTYHM8 (Accessed May 27, 2026).

[B42] MaddyEats (2023). A tub of hot chocolate with chocolate eggs, chocolate bars, chocolate waffle, chocolate cakes, eating. YouTube. Available online at: https://www.youtube.com/watch?v=SYIyj3bIJoI (Accessed February 7, 2026).

[B43] Mahady A. Takac M. De Foe A. (2023). What is autonomous sensory meridian response (ASMR)? A narrative review and comparative analysis of related phenomena. Consciou. Cognit. 109:103477. doi: 10.1016/j.concog.2023.103477 36806854

[B44] Malavika A. (2026). OMAD Diet: What It Is, How It Works, Benefits, and Side Effects | PharmEasy. Mumbai: PharmEasy Blog. Available online at: https://pharmeasy.in/blog/omad-diet-what-it-is-how-it-works-benefits-and-side-effects/ (Accessed May 26, 2026).

[B45] Poerio G. L. Blakey E. Hostler T. J. Veltri T. (2018). More than a feeling: autonomous sensory meridian response (ASMR) is characterized by reliable changes in affect and physiology. PLoS One 13:e0196645. doi: 10.1371/journal.pone.0196645 29924796 PMC6010208

[B46] Rodgers R. F. Slater A. Gordon C. S. McLean S. A. Jarman H. K. Paxton S. J. . (2020). A biopsychosocial model of social media use and body image concerns, disordered eating, and muscle-building behaviors among adolescent girls and boys. J. Youth Adolescence 49, 399–409. doi: 10.1007/s10964-019-01190-0 31907699

[B47] Rubin A. M. (2009). “Uses-and-gratifications perspective on media effects,” in Media Effects (New York, NY: Routledge), 181–200. doi: 10.4324/9780203877111-14

[B48] Sanskriti S. Guglani I. Joshi S. Anjankar A. (2023). The spectrum of motivations behind watching mukbang videos and its health effects on its viewers: a review. Cureus 15:e44392. doi: 10.7759/cureus.44392 37786568 PMC10541680

[B49] Schwegler-Castañer A. (2018). At the intersection of thinness and overconsumption: the ambivalence of munching, crunching, and slurping on camera. Feminist Media Stud. 18, 782–785. doi: 10.1080/14680777.2018.1478694

[B50] Song H. (2018). The making of microcelebrity: afreecatv and the younger generation in neoliberal South Korea. Soc. Media Soc. 4:205630511881490. doi: 10.1177/2056305118814906

[B51] Stake R. (1995). The Art of Case Study Research. [online]. SAGE Publications Ltd. Available online at: https://uk.sagepub.com/en-gb/eur/the-art-of-case-study-research/book4954 (Accessed May 26, 2026).

[B52] Strand M. Gustafsson S. A. (2020). Mukbang and disordered eating: a netnographic analysis of online eating broadcasts. Cult. Med. Psychiatry 44, 586–609. doi: 10.1007/s11013-020-09674-6 32277331 PMC7497418

[B53] The Philippine Daily Inquirer/Asia News Network (2024). ‘Food pornography': Philippines eyes ‘mukbang' ban after food vlogger's death. [online] *The Straits Times*. Available online at: https://www.straitstimes.com/asia/se-asia/food-pornography-philippines-eyes-mukbang-ban-after-food-vlogger-s-death (Accessed May 26, 2026).

[B54] TOI World Desk (2024). 24-year-old Chinese Influencer Pan Xiaoting Dies During Livestream Mukbang Session. Mumbai: The Times of India. Available online at: https://timesofindia.indiatimes.com/world/china/24-year-old-chinese-influencer-pan-xiaoting-dies-during-livestream-mukbang-session/articleshow/111952016.cms (Accessed May 26, 2026).

[B55] World Health Organization (2025). WHO Report on the Global Tobacco Epidemic, 2025: Warning About the Dangers of Tobacco. Geneva: World Health Organization.

[B56] World Health Organization (2019). ICD-11. Geneva: World Health Organization. Available online at: https://icd.who.int/en/ (Accessed May 26, 2026).

[B57] World Medical Association (2024). WMA Declaration of Helsinki – Ethical Principles for Medical Research Involving Human Participants [Online]. Ferney-Voltaire: WMA - the World Medical Association. Available online at: https://www.wma.net/policies-post/wma-declaration-of-helsinki/ (Accessed May 26, 2026).

[B58] Xu J. Qu L. Zhang G. (2022). Governing social eating (chibo) influencers: policies, approach and politics of influencer governance in China. Policy Internet 14, 525–540. doi: 10.1002/poi3.318

[B59] Yin R. K. (2018). Case Study Research and Applications: Design and Methods, 6th edn. Los Angeles: SAGE Publications.

[B60] YouTube. (2018). Saapattu Raman. Available online at: https://www.youtube.com/channel/UChahm6WYl7bD1uWfNEFqcZw (Accessed February 7, 2026).

[B61] YouTube. (2019). Harmful or dangerous content policy: YouTube community guidelines [Online] YouTube.Available online at: https://www.youtube.com/watch?v=RzxSvr_vp1o (Accessed 1 February 2025).

